# Super‐Toughness Carbon Nanotube Yarns by Bio‐Inspired Nano‐Coiling Engineering

**DOI:** 10.1002/advs.202400460

**Published:** 2024-04-23

**Authors:** Young Shik Cho, Jae Won Lee, Yeonsu Jung, Ji Yong Park, Jae Seo Park, Sang Min Kim, Seung Jae Yang, Chong Rae Park

**Affiliations:** ^1^ Institute of Advanced Composite Materials Korea Institute of Science and Technology (KIST) Wanju 55324 Republic of Korea; ^2^ Department of Materials Science & Engineering and Research Institute of Advanced Materials Seoul National University Seoul 08826 Republic of Korea; ^3^ Composite Research Division Korea Institute of Materials Science (KIMS) Changwon 51508 Republic of Korea; ^4^ Department of Chemistry & Chemical Engineering Education and Research Center for Smart Energy and Materials Inha University Incheon 22212 Republic of Korea

**Keywords:** bioinspired, bundle engineering, carbon nanotube, super‐toughness, yarn

## Abstract

Lightweight structural materials are commonly used as effective fillers for advanced composites with high toughness. This study focused on enhancing the toughness of direct‐spun carbon nanotube yarns (CNTYs) by controlling the micro‐textural structure using a water‐gap‐based direct spinning. Drawing inspiration from the structural features of natural spider silk fibroin, characterized by an α‐helix in the amorphous region and β‐sheet in the crystalline region, multiscale bundles within CNTYs are reorganized into a unique nano‐coil‐like structure. This nano‐coiled structure facilitated the efficient dissipation of external mechanical loads through densification with the rearrangement of multiscale bundles, improving specific strength and strain. The resulting CNTYs exhibited exceptional mechanical properties with toughness reaching 250 J g^−1^, making them promising alternatives to commercially available fibers in lightweight, high‐toughness applications. These findings highlight the significance of nano‐coiling engineering for emulating bio‐inspired micro‐textural structures, achieving remarkable enhancement in the toughness of CNTYs.

## Graphical abstract

This study focuses on enhancing the toughness of direct‐spun carbon nanotube yarns (CNTYs) by controlling the micro‐textural structure using a water‐gap‐based direct spinning. Drawing inspiration from the structural features of α‐helix and β‐sheet within natural spider silk fibroin, multiscale bundles are reorganized into a unique nano‐coil‐like structure, resulting in the production of CNTYs with super‐toughness of 250 J g^−1^.

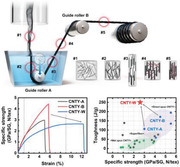

## 1 Introduction

Lightweight structural materials, e.g., carbon fibers and superfibers, are pivotal in advancing modern equipment and enhancing engine efficiency across diverse industries, including aerospace and automotive. Despite significant progress in developing high‐strength materials, realizing high toughness remains a paramount challenge for lightweight structural applications. Carbon nanotubes (CNTs) have attracted considerable attention as a highly promising material owing to their exceptional mechanical properties, with a theoretical modulus and strength reaching as high as 1 TPa and 77 GPa (g cm^−3^)^−1^ [= N tex^−1^],^[^ ^1^ ^]^ respectively. The concept of a CNT yarn (CNTY),^[^ ^2^ ^]^ comprising CNTs overlapping along the axial direction, has been proposed to actualize their outstanding properties and harness the exceptional attributes of CNTs.

Strength and toughness are the two most critical mechanical properties of structural materials. Strength refers to the ability of a material to resist deformation, while toughness represents the resistance to fracture. Typically, these properties are mutually exclusive in most materials. Even in the case of CNTYs, several studies have indicated that the strength and stiffness of the yarns increase as the densification of CNT bundles improves while the strain and toughness decrease because of the reduced load‐bearing capacity resulting from the reorientation of CNT bundles.^[^ ^3^, ^4^ ^]^ Various techniques have been used to fabricate CNTYs, including drawing from a CNT forest,^[^ ^5^ ^]^ direct spinning from a furnace where CNTs are grown,^[^ ^4^, ^6^, ^7^, ^8^ ^]^ and wet spinning of a liquid crystalline CNT solution.^[^ ^9^, ^10^, ^11^, ^12^, ^13^, ^14^ ^]^ In conjunction with these techniques, several additional processes, such as solvent densification, twisting, or a combination of both, have been explored to enhance the properties of as‐spun CNTYs.^[^ ^4^, ^15^, ^16^ ^]^ All preceding studies focused primarily on enhancing the strength of CNTY, rather than improving toughness. These studies often led to decreases in strain and toughness. The pursuit of developing a super‐toughness fiber based on CNTY, which requires high strength and strain, remains an unexplored realm within the field.

**Figure 1:**
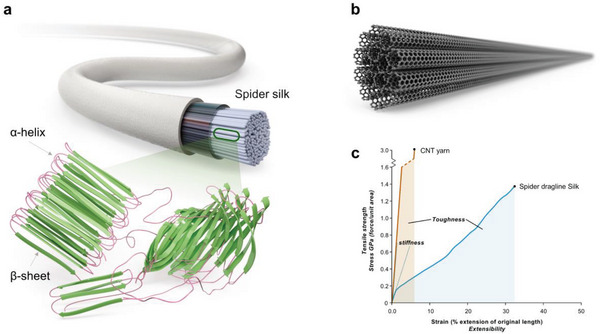
Comparison of the multiscale micro‐textural structures of natural silk fibroin and CNTYs. Schematic diagram of the microstructure of a) natural spider silk fibroin and b) CNTY. c) stress‐strain curve of spider silk and CNTY.

Most natural polypeptide fibrils, including silk fibroins, exhibit a structural composition comprising an amorphous region characterized by an α‐helix structure and a crystalline region characterized by a β‐sheet structure.^[^ ^17^ ^]^ The fundamental mechanistic principle behind these fibrils, which involves the development of an amorphous region to enhance toughness while maintaining a crystalline region to reinforce strength, has been reported.^[^ ^18^, ^19^ ^]^ For example, spider silk, which incorporates both regions, demonstrates higher toughness than Kevlar fiber, which features developed crystalline regions but lacks an amorphous region because of its stiff backbone.^[^ ^20^ ^]^ Given the similarity between the multiscale bundle structure within CNTYs and the microstructure of natural polypeptide fibrils (**Figure** **1**a), it is essential to concentrate efforts on constructing both multiscale bundles with an α‐helix‐like orientation and a β‐sheet‐like arrangement to enhance toughness. Most post‐treatments eventually produce a huge, β‐sheet‐like bundle, but the toughness was not maximized because of the inevitable alignment of CNT bundles far from the helix structure.

This paper presents a novel and environmentally friendly approach for fabricating super‐toughness CNTYs, taking inspiration from the strengthening mechanism observed in natural fibrils. A multiscale bundle structure connected by a nano‐coil‐like bundle was engineered using a water gap coupled with an additional guide roller during the direct spinning process. This engineering strategy resulted in the highest reported toughness for CNTYs, comparable to the performance of existing benchmarks. In particular, the toughness of the CNTYs was enhanced significantly to 250 J g^−1^ while maintaining a high specific strength of 2.6 N tex^−1^. This toughness surpassed that of spider silk, which is renowned for its exceptional toughness (≈100 J g^−1^). The implications of these findings are profound because they demonstrate the feasibility of tailor‐fitted enhancement of the toughness of CNTYs by engineering multiscale bundles inspired by the microstructure of natural polypeptide fibrils. This improvement can be realized using a simple, environmentally friendly, and practical fabrication process.

## 2 Results and Discussion

### 2.1 Bio‐Inspired Micro‐Texture in CNTY

Polypeptides, as a prominent constituent of natural fibrils, exhibit a distinct structural arrangement characterized by α‐helices and β‐sheets, facilitated by robust hydrogen bonding between polymer chains. The α‐helix is the primary building block within the glassy amorphous region, while the β‐sheet is the main building block within the crystalline region.^[^ ^17^ ^]^ These regions play a critical role in determining the mechanical properties of natural fibrils. The simultaneous enhancement of both strength and strain leads to the achievement of high toughness. In particular, natural spider silk has emerged as an exemplar of high‐toughness fiber, owing to its strength augmentation through the internal crystalline region and strain via the amorphous region.^[^ ^19^, ^21^ ^]^ The exceptional strain of spider silk fibroin can be attributed to the spring‐like structure of α‐helices within the amorphous region. In contrast, its high strength stems from the dense arrangement of β‐sheets within the crystalline region, as shown in Figure 1a.

Inspired by the concept of natural silk fibroin, a new approach has been developed to engineer the multiscale bundle structure within CNTYs. Just as the β‐sheet‐based crystalline section in silk fibroin contributes to its tensile strength, a dense and well‐oriented multiscale bundle, as depicted in Figure 1b, is advantageous for enhancing the tensile strength of CNTY. On the other hand, this stiff structure has limitations because it leads to decreased toughness owing to the lower strain than spider silk, as shown in the stress–strain curve in Figure 1c.^[^ ^7^, ^21^ ^]^ Instead, achieving a high‐toughness CNTY necessitates the development of a less‐oriented, coil‐structured multiscale bundle. Consequently, a specialized engineering process is required to adjust the degree of orientation while aggregating each bundle. In this study, the orientation of CNTs was controlled during the direct spinning process without deforming the multiscale bundle, achieving a CNTY with exceptionally high toughness.

### 2.2 Nano‐Coiling Engineering of the Multiscale Bundle in CNTY During Direct Spinning

**Figure 2:**
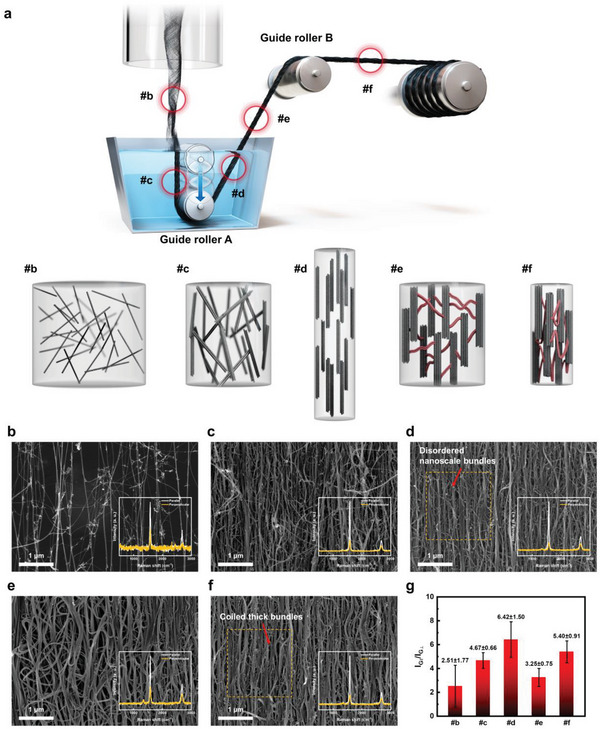
Development of the multiscale micro‐textural structure of CNT assemblies during the fabrication process. a) Schematic diagram of the development of the multiscale micro‐textural structure at position #B‐F on the direct spin‐line. SEM images of radial sections of the CNT assemblies at each position: b) a formation of CNT web in the furnace, c) coagulation of CNT web into CNTY at the surface of the water bath, d) a stretch of CNTY in the water bath, e) a rapid shrink of CNTY above the water bath, and f) densification of CNTY through the guide roller B. (inset: polarized Raman spectra of the corresponding position., *n* = 5) g) The G peak intensity ratio (I_G∥_/I_G⊥_) of the polarized Raman spectra at position #b‐f.

In the direct spinning process, the double‐walled CNTs (DWCNTs) growing in a furnace readily assemble into a web with an aerogel‐like structure. This web is then pulled through guide roller A, immersed in a water bath, and further guided using an additional guide roller B, aided by a take‐up roller operating at 5 m min^−1^. In particular, guide roller A in this study was positioned ≈3 cm deeper than in previous reports, resulting in a water‐gap difference. The synthesized CNTY produced using this modified setup is denoted as CNTY‐W. As part of the bundle‐engineering process, the orientation of the CNT bundle was adjusted during the spinning process, utilizing the water gap, as illustrated in **Figure** **2**a.

The direct spinning method provides a way to control the microstructure of the CNTY precisely based on the conditions within and outside the water bath. Within the water bath, bundle engineering was used to isolate the bundles from each other, resulting from the CNTYs swelling and expanding. In contrast, bundle engineering is applied to densify the bundles outside the water bath because of the CNTYs contract. The swollen state needs to be maintained because the bundles within the dried CNTYs are intricately entangled and have limited degrees of freedom, making them challenging to manipulate. Introducing a water gap plays a crucial role in extending the path of the CNTYs within the water bath, facilitating efficient bundle engineering. Therefore, during the spinning process, the orientation of the CNT bundle in the CNTY changes continuously, which significantly influences the properties of the resulting yarn. The microstructure and orientation at each process stage (Figure 2b–f) and specific positions were analyzed by the scanning electron microscopy (SEM) and the polarized Raman spectroscopy. At the first position (#b), known as the air gap, just before entering the water bath, the majority of the CNT bundles exist without significant aggregation (Figure 2b). The orientation of the CNTs, measured by the G peak intensity ratio (I_G∥_/I_G⊥_) in the polarized Raman spectrum,^[^ ^8^ ^]^ was ≈2.51 ± 1.77 (*n* = 5). At the second position (#c), corresponding to the water gap, the hydrophobic nature of the CNTs acts as a driving force, leading to the aggregation of CNT bundles within the web. This improves the CNT orientation to an intensity ratio of 4.67 ± 0.66 (Figure 2c, *n* = 5). At the third position (#d), immediately after passing guide roller A in the water bath, water swelling within the CNTY intensifies, and tension from guide rollers A and B generates a wet‐drawing effect. The freely movable bundle within the swollen CNTY undergoes further aggregation and reassembly. Consequently, the length of the multiscale bundle increases, leading to an enhanced CNT orientation of 6.42 ± 1.50 (Figure 2d, *n* = 5), reaching the highest point in the process. At the fourth position (#e), immediately after the CNTY exits the water bath, the rapid contraction of the CNTY due to the absence of surrounding water causes the extended multiscale bundle to fold in the axial direction, resembling a coiled fiber. This folding leads to a decrease in the CNT orientation to ≈3.25 ± 0.75 (Figure 2e, *n* = 5). Finally, at the last position (#f), immediately after passing through guide roller B, tension‐induced bundle aggregation fixes the folded structure to form an α‐helix‐like bundle and densifies the structure to form a β‐sheet‐like bundle (Figure 2f). In Figure 2d, the ordered regions are connected by thin, disordered bundles, whereas in Figure 2f, these ordered regions are connected by thicker bundles twisted into a coiled structure. In this step, the orientation of the CNT increases to 5.40 ± 0.91 (*n* = 5). Figure 2g summarizes the overall orientation of the CNT at each stage. The introduction of the water gap intensifies the processes at #c and #d, resulting in a unique structure, as observed in Figure 2e.

The improved strain characteristics are essential for realizing the super‐toughness CNTY. Inspired by the manufacturing method of CNT strain sensors or actuators, which involves lowering the orientation of CNTs by coiling on a macro‐scale, the microstructures within the CNTY were wrinkled. They reassembled into a spring‐like structure during the manufacturing process. Some of the CNTYs that exhibit significantly lower strength may show coiled (or helical) structures due to alignment issues and low density. On the other hand, CNTYs that do not intentionally form coil structures have well‐organized bundle arrangements and generally exhibit above‐average strength. However, the former case presents a significant decrease in strength, while the latter has limitations such as low toughness and strain. Thus, “coiled structure” should be defined as intentionally twisted thicker bundles with diameters ≈50–100 nm, which is the key factor for both strength and toughness.

**Figure 3:**
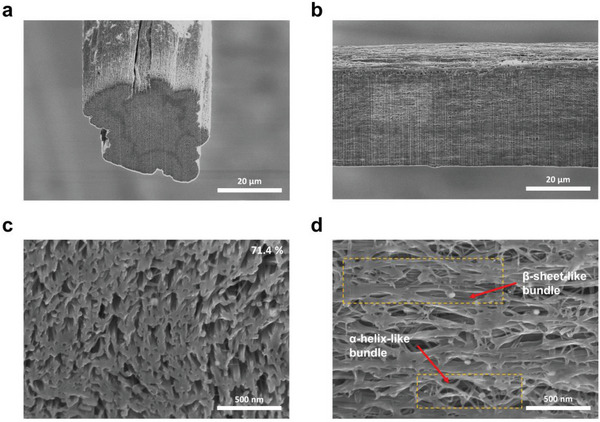
Nano‐coiled micro‐textural structure of multiscale bundles within the CNTY‐W. SEM images of FIB cut a,c) cross‐sections and b,d) radial sections of CNTY‐W in (a, b) low and c,d) high magnification.

The CNTY‐W was synthesized with a diameter and linear density of 32 µm (**Figure** **3**a,b) and 0.156 tex [= g km^−1^], respectively. The yarn was composed of DWCNTs with an outer diameter of ≈4 nm (Figure S1, Supporting Information). Thermogravimetric analysis (TGA) revealed the composition of CNTY‐W, with 77.0 wt.% of DWCNTs,^[^ ^22^ ^]^ 9.2 wt.% of amorphous carbon, and 14.8 wt.% of residual Fe catalyst (Figure S2a, Supporting Information). A high amount of DWCNTs in the yarn contributes to its mechanical performance by providing a load‐bearing unit. Raman spectroscopy analysis showed that the intensity ratio of the G‐band to the D‐band (I_G_/I_D_) was ≈37.7 ± 9.7 (Figure S2b, Supporting Information, *n* = 5), suggesting that the elementary CNTs are highly crystalline. The bundle structures determine the properties of CNTY‐W owing to the high quality of the elementary CNTs.

The assembly and orientation behavior of CNTY‐W were analyzed by focused ion beam (FIB) analysis on cross‐sections (Figure 3a,c) and radial sections (Figure 3b,d) of the yarn. The effective cross‐sectional area of CNTY‐W, representing the proportion of the area occupied by the multiscale bundles to the total area, was 70.3 ± 3.5% (*n* = 5, Figure 3c; Figure S3, Supporting Information). A densely packed multiscale bundle made the CNTY‐W more effective in dissipating the tensile deformation energy that is imposed on it. This is crucial for enhancing the overall mechanical performance and toughness of the material. Furthermore, a densely packed bundle of ≈140 nm was formed by engineering the CNT bundles, resembling the β‐sheet of a polypeptide fibril. Furthermore, a bundle, ≈60 nm in size, connected the densely packed bundles, similar to the α‐helix of a polypeptide fibril. This arrangement resulted in a nano‐coiled structure according to the radial‐section analysis (Figure 3d). The nano‐coiled structure facilitated the reorientation of the interconnected β‐sheet‐like bundles and efficiently dissipated the external loads. The microstructure in Figure 3 showed that CNTY‐W consists of a well‐aligned β‐sheet‐like bundle as a main frame and an α‐helix‐like bundle connecting the frames in the form of nano‐coil, making it similar to the dual component structure of silk fibroin.

**Figure 4:**
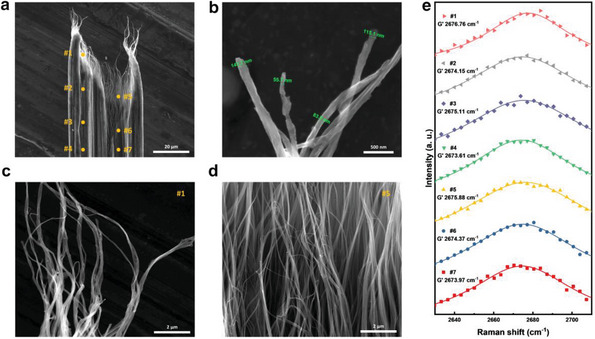
Bundling behavior within CNTY‐W at the fracture surface. SEM images of a) the fracture surface of CNTY‐W with position designation (#1–#7), b) multiscale bundles with variable diameters at the end of the fracture surface, and microstructure of the failure position c) #1, and d) #5. e) G′ Raman band of positions #1 to #7.

Fracture surface analysis of CNTY‐W was conducted to gain insights into the elongation of load‐bearing elements and the failure mechanism of the dual‐structured multiscale bundle within the yarn. The fracture surface of CNTY‐W revealed the release of a nano‐scaled load‐bearing element from a well‐densified β‐sheet‐like bundle, as shown in **Figure** **4**a. Compared to a previous report, the failure section of CNTY‐W lengthened up to 40 from 10 µm, indicating that the load‐bearing bundles within CNTY‐W had undergone additional stretching during the water gap of the spinning process. Furthermore, as shown in Figure 4b, the diameter of the load‐bearing element within the β‐sheet‐like bundle at the failure section was ≈60 nm, corresponding to the diameter of the α‐helix‐like bundle. These results suggest that the building blocks of the α‐helix‐like bundle and β‐sheet‐like bundle within the dual structure are the same and match the primary element responsible for transferring the external load.

Seven points were designated in the failure section (Figure 4a), ranging from the end of the yarn to the body of the yarn. SEM images of positions #1 (Figure 4c) and #5 (Figure 4d), corresponding to the start and end points of the failure section, showed that the majority of released bundles are the primary load‐bearing elements and they remain in significant contact with each other in aligned form. The Raman G′ band of the seven points was analyzed to determine the load transfer efficiency between neighboring CNTs (Figure 4e). Orthogonal electronic dispersion occurs when CNTs are in contact, causing the G′ peak to shift to lower energies. Therefore, the shift in the G′ peak position indicates the extent of the inter‐nanotube contact area.^[^ ^23^ ^]^ The G′ band at the yarn body appeared at ≈2673 cm^−1^, while the G′ band at the end of the yarn appeared at ≈2676 cm^−1^. The similarity in the Raman shift of the G′ band suggests that the degree of contact between the multiscale bundles remains similar regardless of the position within the failure section. The formation of coils by bundles does not refer to the disorderliness of thin bundles, but rather to the intentional twisting of thickly formed bundles with a diameter of ≈50–100 nm. Even though the helix structure and sheet structure are only different in arrangement, the bundle configuration became the same through the water‐gap process as shown in Figure 2. Thus, bundles forming coils would also exhibit G′ bands similar to the ordered regions. When the yarn ultimately fails, similarly sized bundles in both helix‐ and sheet‐like structures dissipated the external tensile load, leading to similar behavior in the G′ band at #1 to #7. These results confirm the similar inter‐bundle CNT and intra‐bundle CNT interactions for the load‐bearing element and support the coincidence of the primary load‐bearing element and the basic building block of the dual assembly structure.

### 2.3 Mechanical Performance of CNTY‐W According to the Load‐Bearing Mechanism of Nano‐Coiled Micro‐Texture

**Figure 5:**
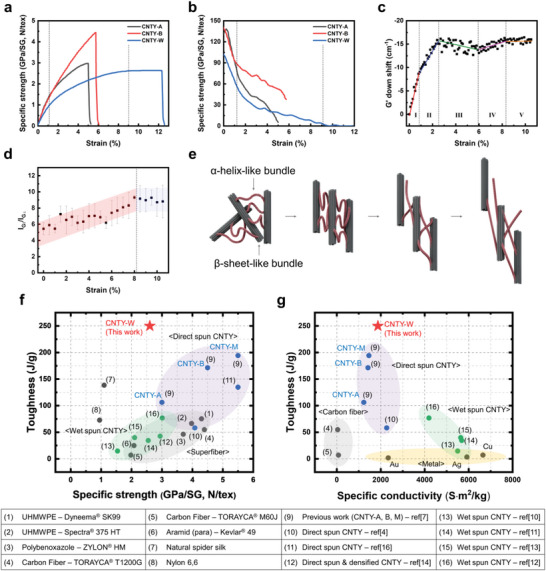
Mechanical properties and analyses of the CNTY‐W. a) Specific stress‐strain curve and b) specific modulus‐strain curve of CNTY‐W (this study, *n* = 10) and CNTY‐A, B (previous study). In situ c) down‐shift of the G′ peak positions (Raman spectra) and d) G peak intensity ratio (polarized Raman spectra) of CNTY‐W during the tensile load (*n* = 3). e) Schematic diagram of the load‐bearing mechanism of nano‐coiled multiscale bundles within the CNTY‐W. Ashby plots of the present CNTY‐W (star) compared to various high‐performance fibers (circle) in terms of the toughness versus the f) specific strength and g) specific electrical conductivity.

The micro‐texture in CNTY‐W was controlled by introducing a water gap in the direct spinning process, forming oriented and disoriented sections. The two incompatible sections created synergy, resulting in remarkable mechanical properties. In particular, CNTY‐W exhibited a tensile strain, specific strength, and specific modulus of 12.5 ± 0.9%, 2.6 ± 0.1 N tex^−1^, and 97.5 ± 8.0 N tex^−1^, respectively, as shown in the stress‐strain curve of **Figure** **5**a (*n* = 10). The toughness of CNTY‐W reached 250 J g^−1^ (*n* = 10) from 170 J g^−1^, surpassing those of benchmark super‐fibers. Compared to the strength‐enhanced yarn CNTY‐B in a previous report,^[^ ^7^ ^]^ which had been spun without a water gap, the specific strength and modulus decreased, while the strain significantly increased. Additionally, compared to the as‐spun yarn CNTY‐A, CNTY‐W maintained a similar specific strength while significantly increasing the strain. This significant improvement in toughness was attributed to the substantially increased strain achieved by the nano‐coiling engineering of multiscale bundles.

The stress‐strain curve in Figure 5a revealed the unique characteristic of CNTY‐W, whereby the yarn can withstand a given strain for an extended duration while maintaining the ultimate tensile strength. This contrasts with CNTY‐A & B in a previous report,^[^ ^7^ ^]^ where the strain at ultimate tensile strength and failure were similar, as shown in Figure 5a. CNTY‐W showed a significant difference (>2%) between these two strains. On the other hand, the modulus‐strain curve in Figure 5b showed that the specific modulus of CNTY‐W could be divided broadly into three distinct features. First, the initial sharp decline indicates an elastic region where the load is transferred primarily through the β‐sheet‐like bundle. The gradual decline indicates a plastic region where the load is transferred dominantly through the α‐helix‐like bundle. This two‐mode decline in the modulus curve indicates the presence of a hierarchical structure composed of two different kinds of bundles. Finally, there is a region where the modulus converges to zero. This last region corresponds to the section where plastic instability occurs. When comparing with CNTY‐A & B from a previous report, it is observed that the presence of β sheet‐like bundles in CNTY‐A, when thickened to form CNTY‐B, leads to a significant increase in tensile strength, up to 4.4 N tex^−1^. Conversely, when some of these β sheet‐like bundles transform into α helix‐like bundles to create CNTY‐W, the tensile strength is almost maintained, while the strain improves up to 12%.

During the plastic deformation of CNTY, there is competition between two phenomena that contribute to its strength: load transfer via bundling behavior and load transfer resulting from the stretching of multiscale bundles. Load transfer via bundling is dominant, resulting in a greater modulus than the stress. On the other hand, plastic instability occurs in CNTY‐W, where the strain exceeds 10%. This leads to a reversal of the inequality between stress and modulus, causing the modulus to converge to zero. The plastic instability results from unstable load transfer at a locally failed site. Caused by the stretching of multiscale bundles, this local failure results from the pullout of an α‐helix‐like bundle. The contact surface between the α‐helix‐like bundle and β‐sheet‐like is the weakest point locally. In the case of load transfer within CNTY‐W, the behavior of the nano‐coiled bundle structure varies with the level of strain. When the strain is below 10%, the nano‐coiled bundles reorientate, similar to the stretching of elastic materials. By contrast, when the strain exceeds 10%, the nano‐coiled bundles undergo stretching similar to the necking of plastic materials from a macroscopic perspective. This stretching is facilitated by the pullout of the primary load‐bearing unit until local failure, ultimately contributing to the observed plastic deformation behavior, as shown in Figure 3b.

The mechanical properties of CNTY are strongly influenced by the assembly of CNTs, which is determined by the characteristics of the load‐bearing mechanism. In the case of CNTY‐W, further analysis was conducted to examine its bundling properties and orientation behavior. The bundling properties of CNTY‐W were examined by the downshift of G′ band from in situ Raman spectroscopy during tensile strain, as shown in Figure 5c (*n* = 3). The extent of bundling of the multiscale bundles is a critical factor affecting the total area of shear failure. The deformation behavior of CNTY‐W exhibited additional zones, including a plastic instability region, in addition to the four zones reported.

A notable characteristic observed in the bundling behavior of CNTY‐W is the widened Zone III in the plastic region. This suggests that the α‐helix‐like bundles within CNTY‐W could transfer external loads efficiently through effective slippage between them. The elongation of α‐helix‐like bundles achieved during yarn spinning played a crucial role in maintaining Zone III by preventing the concentration of weak points, such as the endpoints of multiscale bundles, in a single location when subjected to external loads. Consequently, Zone IV was also widened, leading to the dispersion of partial failure throughout CNTY‐W. According to the weakest link theory, the probability of yarn rupture decreases as the length of fibrils increases. The lengthened multiscale bundles in CNTY‐W contribute to the resistance against catastrophic failure by dispersing partial failures. Furthermore, Raman analysis showed no macroscopic change or peak shift in the bundling behavior was observed in the subsequent plastic instability zone. Hence, the entire pullout of the primary load‐bearing unit, rather than the rupture of the multiscale bundle, occurred during the plastic instability zone. In contrast to the plastic zone, the transfer efficiency of CNTY‐W in the elastic zone showed a shift in the Raman G′ peak of −10.30 cm^−1^/%, starting from 2671.3 cm^−1^. Furthermore, the maximum peak shift was −16.16 cm^−1^, which was slightly lower than that reported elsewhere. Nevertheless, the high toughness observed in CNTY‐W suggests that the orientation behavior of α‐helix‐like bundles contributes to the load‐bearing mechanism.

The orientation behavior of bundles in CNTY‐W was analyzed using in situ polarized Raman spectroscopy (Figure 5d, *n* = 3). Throughout the transition from the initial state to the end of Zone IV, the intensity factor (I_G∥_/I_G⊥_), which is indicative of the orientation of CNTs, exhibited a continuous increase from 5.40 ± 0.91 to 9.16 ± 1.62. In a conventional load‐bearing mechanism, the intensification of bundling tends to accelerate slippage between the bundles, making it challenging for the yarn to achieve a high strain. On the other hand, the section of load transfer via bundling was shifted to a higher strain region because of the efficient load transfer supported by the reorientation of the nano‐coiled bundles. This allowed CNTY‐W to increase its toughness, as observed. Similarly, the orientation of the CNTs remained constant during the plastic instability zone, which is consistent with the behavior observed in bundlings. This indicates the stretching of multiscale bundles within the yarn. Figure 5e summarizes the overall progression of load transfer within the nano‐coiled multiscale bundle.

In summary, the mechanical properties are determined by the load‐bearing mechanism each phase specializes in. In the case of the β‐sheet phase, load transfer is achieved by maximizing frictional force through bundle densification. On the other hand, the α‐helix phase enhances load transfer by rearranging bundles to improve frictional force. As a result, the β‐sheet phase contributes to the strength of the CNTY, while the α‐helix phase affects its strain. In CNTYs where the β‐sheet phase predominates, such as CSA‐spun CNT yarn,^[^ ^10^, ^11^, ^12^, ^13^ ^]^ the strength improves but the yarn becomes brittle, leading to decreased strain. The dense packing and axial alignment of β‐sheet phases within the yarn greatly enhance its strength through bundle densification but hinder rearrangement, resulting in reduced strain and toughness. Conversely, in CNTYs where the α‐helix phase dominates, such as directly‐spun CNT yarn,^[^ ^7^, ^16^ ^]^ the toughness improves while the strength decreases. Although the α‐helix phase, which allows freer movement under external forces, leads to less densification of bundles and a smaller increase in strength compared to the β‐sheet phase, its ease of rearrangement of bundles enhanced the toughness of CNTY.

These findings indicate the relationship between bundling, orientation behavior, and mechanical properties, providing insights into the role of assembly in determining the overall performance and failure mechanisms of CNTY‐W. The reorientation of bundles and efficient load transfer contribute to the enhanced toughness and strain capabilities of CNTY‐W, highlighting the importance of these factors in the design and engineering of high‐performance materials.

## 3 Conclusion

The specific mechanical performance of CNTY‐W fabricated in this study is exceptional, surpassing previously reported CNTYs and many commercialized high‐performance fibrous materials (Figure 5f). The toughness of CNTY‐W alone exceeded that of Kevlar aramid fibers, which are the market‐dominating high‐performance organic fibers. Furthermore, CNTY‐W can be produced in a straightforward and environmentally friendly manner and exhibits outstanding mechanical performance (specific tensile strength and toughness of 2.6 N tex^−1^ and 250 J g^−1^), respectively. The strain at failure of CNTY‐W reached 12.5%, which outperformed other high‐performance fibers. This high strain capacity enables easy performing during composite manufacturing without yarn breakage. CNTY‐W possesses a specific electrical conductivity of 1.87 × 10^3^ Sm^2^ kg^−1^ (*n* = 25), which is sufficiently high to be comparable with CSA‐doped direct‐spun CNTYs, as shown in Figure 5g. This electrical conductivity was attributed to the elongated multiscale bundle structure achieved through water gap‐based nano‐coiling engineering. Generally, commercialized materials lack high toughness and specific electrical conductivity. By contrast, the exceptional mechanical and electrical performance of CNTY‐W distinguishes it as a remarkable material with unique properties.

This study presents a novel approach to producing high‐toughness CNTYs through water‐gap engineering in the direct spinning process. The exceptional mechanical performance of these CNTY was attributed to the well‐developed multiscale nano‐coiled structure, drawing inspiration from the α‐helix structure found in the amorphous region and the β‐sheet structure found in the crystalline region of spider silk fibroin. Considering that most of the micro‐texture within the CNTY is determined by the processing conditions, the multiscale bundles into a nano‐coil‐like structure were reorganized by controlling the drawing ratio through the water gap during direct spinning. The feasibility of producing high‐toughness CNTYs with superior mechanical performance was demonstrated by incorporating the concept of natural fibril micro‐texture into the manufacturing process.

## 4 Experimental Section

### Materials and the Direct Spinning Conditions for the Fabrication of DWCNT Yarns

DWCNTs were synthesized using a floating catalyst chemical vapor deposition method in a vertical alumina tube reactor, according to the procedure described elsewhere.^[^ ^7^, ^24^ ^]^ The DWCNTs obtained from the synthesis process were spun directly into carbon nanotube yarns (CNTYs) by passing the assembled aerogel‐like web through a water bath. In this process, guide‐roller A was positioned 4 cm below the water surface, which differed from previous reports where it was placed just below the surface at a 1 cm depth.^[^ ^7^ ^]^ The ≈3 cm difference in the position of guide‐roller A was called the “water gap.” The water gap was set to 3 cm to match the drawing ratio of this work to the previous work. Guide‐roller B was placed directly above the water bath, and a take‐up roller took up the CNTY sample at a 5 m min^−1^ winding speed. The CNTY sample obtained after passing through the water gap and guide rollers A and B was labeled CNTY‐W.

### Mechanical and Structural Characterizations of CNTYs

The tensile performance of the CNTYs was tested on a tensile tester (TST350, Linkam) at a gauge length of 10 mm and a strain rate of 3 mm min^−1^. The linear density of the given CNTY was determined by measuring the weight of 20 m of CNT yarn. In situ, Raman spectroscopy was carried out while the CNTY was strained with a gauge length of 30 mm on a tensile stage set on the sample stage of the Raman spectroscope. The CNT nanostructure was examined by field‐emission transmission electron microscopy (FETEM, JEM‐3000F, JEOL, Japan) and Raman spectroscopy (RAMANplus, Nanophoton) with a 532 nm laser. The purity of the CNTs was determined by thermogravimetric analysis (TGA; SDT‐Q600, TA Instruments) in air condition with a heating rate of 10 °C min^−1^. The cross‐ and radial‐sections of the CNTYs were characterized by field‐emission scanning electron microscopy (FESEM, SUPRA 55VP, Carl Zeiss, Germany) after cutting with a focused ion beam (FIB; Helios 650, FEI). The ImageJ program (National Institutes of Health, USA, ver. 1.51j8) was used to calculate the effective cross‐sectional area of CNTY on the basis of their cross‐sectional SEM images.^[^ ^16^ ^]^

## Conflict of Interest

There are no conflicts of interest to declare.

## Author Contributions

Y.S.C. conceived and designed the experiments. Y.S.C, J.W.L., and Y.J. contributed to the synthesis of CNTYs using the direct spinning method. Y.S.C., J.Y.P., J.S.P., and S.M.K. characterized CNTYs and wrote the paper. S.J.Y. and C.R.P. supervised the project. All the authors discussed the results and contributed to the preparation of the paper.

## Supporting information

Supporting information

## Data Availability

The data that support the findings of this study are available from the corresponding author upon reasonable request.
