# Supplementary material for: Super‐Toughness Carbon Nanotube Yarns by Bio‐Inspired Nano‐Coiling Engineering
Source: Adv Sci (Weinh). 2024 Apr 23;11(25):2400460. doi: 10.1002/advs.202400460 (PMC11220680; doi:10.1002/advs.202400460)
Supplement: Supplementary file 1 — Supporting information [file ADVS-11-2400460-s001.pdf]

ADVANCED SCIENCE
Open Access

Supporting Information

for *Adv. Sci.*, DOI 10.1002/advs.202400460

Super-Toughness Carbon Nanotube Yarns by Bio-Inspired Nano-Coiling Engineering

Young Shik Cho, Jae Won Lee, Yeonsu Jung, Ji Yong Park, Jae Seo Park, Sang Min Kim, Seung Jae Yang and Chong Rae Park**

Suppplemental information

Super-toughness carbon nanotube yarns by bio-inspired nano-coiling engineering

Young Shik Cho, Jae Won Lee, Yeonsu Jung, Ji Yong Park, Jae Seo Park, Sang Min Kim, Seung Jae Yang and Chong Rae Park**

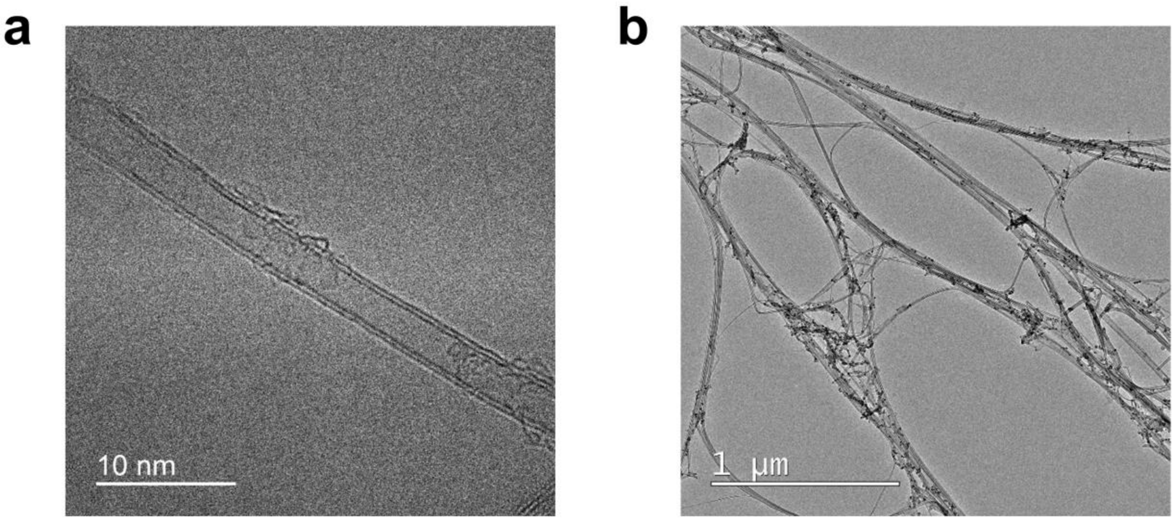

Figure S1. Structure of individual CNT and its assembly. TEM images of (a) as-synthesized individual double-walled CNT and (b) network structure of DWCNTs.

*Corresponding Author.

E-mail address: crpark@snu.ac.kr (C. R. Park) sjyang@inha.ac.kr (S. J. Yang)

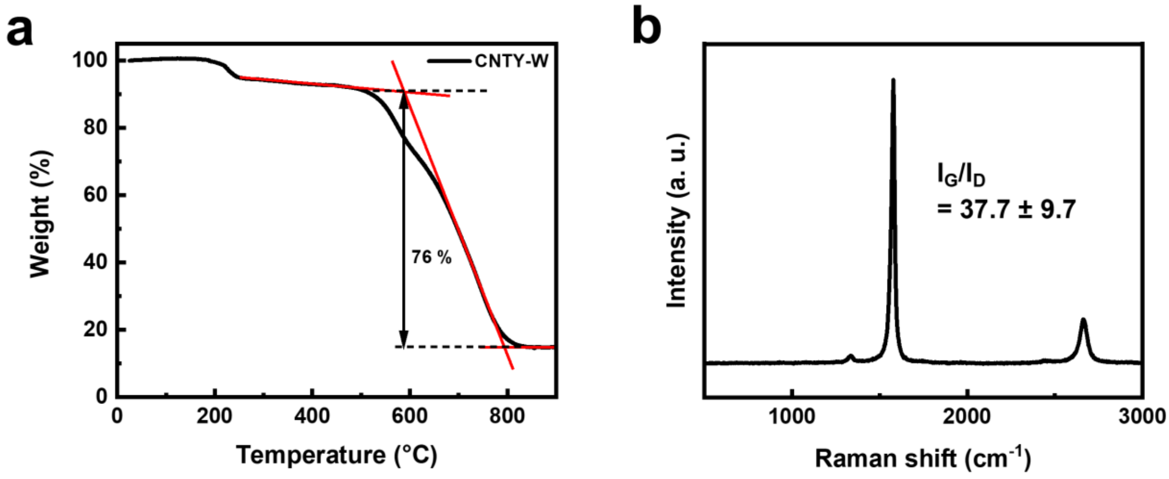

Figure S2. Properties of as-directly spun CNTYs. (a) TGA thermogram and (b) Raman spectra (*n* = 5) of DWCNT yarns.

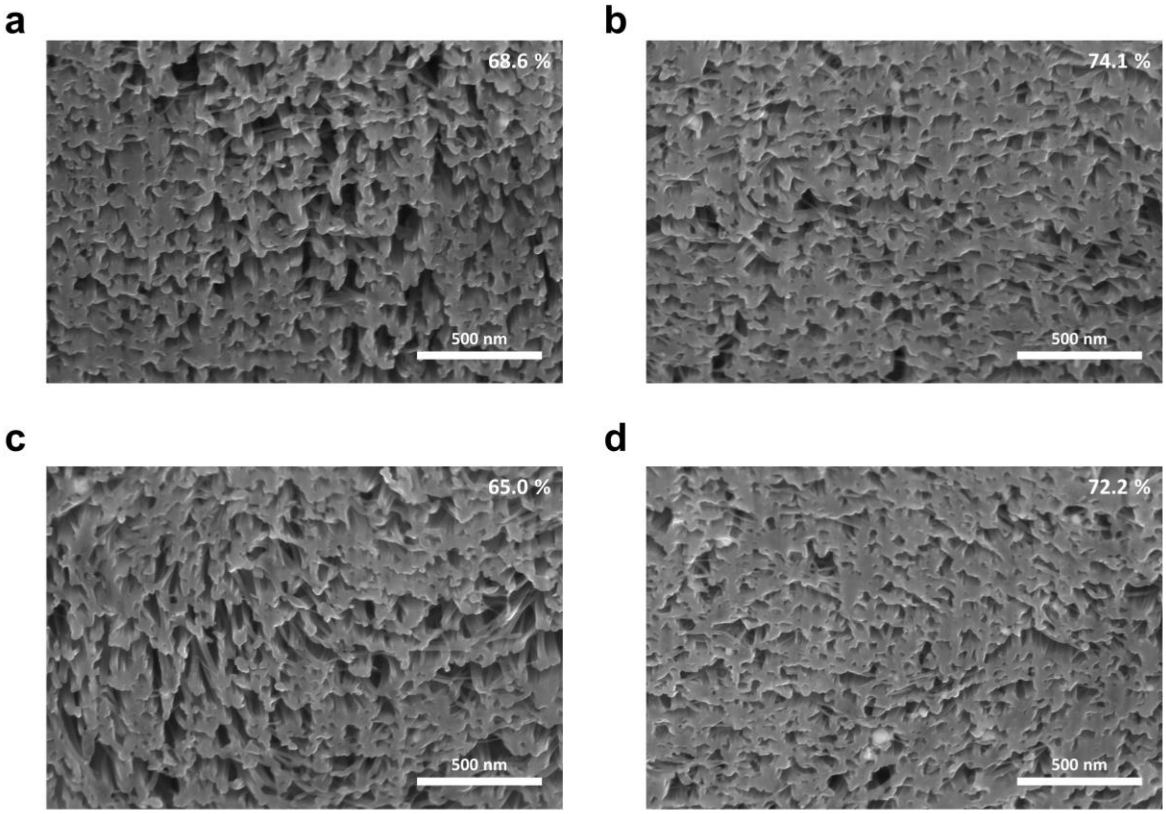

Fig. S3. Various micro-textural structure of multiscale bundles within the CNTY-W. (a-d) SEM images of FIB cut cross-sections of CNTY-W with various effective cross-section area.
